# Interterminal Truck Routing Optimization Using Deep Reinforcement Learning

**DOI:** 10.3390/s20205794

**Published:** 2020-10-13

**Authors:** Taufik Nur Adi, Yelita Anggiane Iskandar, Hyerim Bae

**Affiliations:** Department of Industrial Engineering, Pusan National University, Busan 46241, Korea; taufiknuradi@pusan.ac.kr (T.N.A.); yelita_iskandar@pusan.ac.kr (Y.A.I.)

**Keywords:** interterminal truck routing, deep reinforcement learning

## Abstract

The continued growth of the volume of global containerized transport necessitates that most of the major ports in the world improve port productivity by investing in more interconnected terminals. The development of the multiterminal system escalates the complexity of the container transport process and increases the demand for container exchange between different terminals within a port, known as interterminal transport (ITT). Trucks are still the primary modes of freight transportation to transport containers among most terminals. A trucking company needs to consider proper truck routing planning because, based on several studies, it played an essential role in coordinating ITT flows. Furthermore, optimal truck routing in the context of ITT significantly affects port productivity and efficiency. The study of deep reinforcement learning in truck routing optimization is still limited. In this study, we propose deep reinforcement learning to provide truck routes of a given container transport order by considering several significant factors such as order origin, destination, time window, and due date. To assess its performance, we compared between the proposed method and two approaches that are used to solve truck routing problems. The experiment results showed that the proposed method obtains considerably better results compared to the other algorithms.

## 1. Introduction

**Figure 1:**
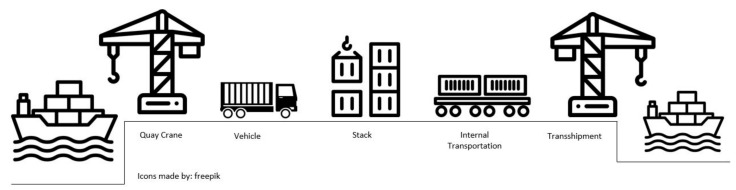
Process of unloading and loading a ship, adopted from [4].

Currently, shipping containers have become ubiquitous and widely adopted by the business world as a standard method of efficient freight transportation. In August 2018, the United Nations Conference on Trade and Development (UNCTAD) reported that global containerized trade from January 2017 to June 2018 reached 10.7 billion tons, a 6.4% increase from the previous year [1]. The immense growth of global containerized trade has created many challenges for ports worldwide. Most of the major ports, such as Shanghai, Rotterdam, Hong Kong, and Singapore, have expanded their ports by developing more terminals to satisfy the demand and maintain customer satisfaction. The need for developing new container terminals requires investments in more port equipment and facilities such as berths, cranes, straddle carriers, terminal operators, and internal trucks. To provide acceptable service levels for importers and exporters, these infrastructural facilities and services need to be addressed and considered by port authority. Hu et al. [2] stated that a multiterminal system increases the complexity of the container transport process. Figure 1 illustrates the process of unloading and loading containers from/to a ship at a typical modern container terminal. The unloading and loading process can be divided into different subprocesses. Ideally, when a vessel arrives at a terminal in a port, the export container is loaded for deep-sea transport, and import containers are unloaded from the ship and directly transported to the customer. Some containers can be stored temporarily at the stack, transferred to different transportation modes, or exchanged between terminals. The movement of containers among terminals is known as interterminal transport (ITT). Tierney et al. [3] defined ITT as any land and sea transportation that moves containers and cargoes between organizationally separated areas within a seaport. This transportation includes container transport among container terminals, empty container depots, logistics facilities (e.g., warehouses, container freight stations), dedicated transport terminals (e.g., barge and rail terminals, dry ports), and administrative facilities (e.g., container screening and custom clearance). ITT can be avoided by optimizing the schedule of container vessels that transship containers to arrive at the same terminal and providing all the required logistics components of a port in the same location. However, a high level of ITT is required for some ports, such as mid- to large-sized ports. Owing to the limitations of port space, it is not always possible to build all the dedicated transport terminals and their connections in one place. Therefore, ITT plays an essential role in improving the link among terminals and can compensate for the differences in infrastructure between terminals. Complex transportation formed by ITT must be handled efficiently by port authorities; otherwise, it can become a significant cause of ITT-related costs, source of errors, and delays. Developing an efficient ITT system can be beneficial to port authorities by minimizing transport delays, transport time and expenses, handling times, and avoiding severe traffic congestion that influences both the productivity and efficiency of the ITT operations.

A port provides alternatives such as roads, rails, and barge services to transport containers between its terminals and facilities. However, according to Islam [5], trucks are still a dominant mode of cargo transportation in many ports and are projected to continue in the future. For example, in the case of the Port of Rotterdam, trucks have the maximum market share, which reaches 60% of the current modal split. The remaining percentage is divided between barge and rail transportation, with barge transportation having more than twice the share of rail transportation. A similar example was also observed in Europe, according to Nicodème et al. [6], during the period from 1995 to 2014; the European hinterland market share for road transportation increased by approximately 3.6%, representing the highest market share of 71.9%, and the demand for rail decreased by approximately 8%. From these statistical data, the truck still dominates the market. In the context of ITT, a truck operation without a proper truck schedule and route planning cause unnecessary operational costs and environmental issues. Heilig and Voß’s study [7] showed that optimizing vehicle routes combined with scheduling approaches provides necessary decision support for terminal operators. It also offers many opportunities to reduce operational costs, improve customer satisfaction, and reduce environmental impact. In most studies, vehicle routing optimization had the objective of minimizing the overall cost related to the use of trucks in the selected routes. Some researchers have attempted to address truck routing optimization in the context of ITT to minimize the overall cost related to the use of trucks. However, most of them used a mathematical model and metaheuristic approaches. To the best of our knowledge, the use of the reinforcement learning-based approach remains limited. Therefore, this study makes the following contributions:

(1) We propose a deep reinforcement learning approach to provide feasible truck routes that minimize the overall cost related to the use of trucks by considering essential requirements such as time windows.
(2) The learning model produced after the deep reinforcement learning training process can be used to provide feasible truck routes for ITT, which has similar characteristics, in terms of the number of container terminals, within short computational times. These performance characteristics are critical in the context of real-time, real-world applications.
(3) To evaluate the proposed method, we conduct computational experiments using artificially generated data and compare it with two metaheuristic methods: simulated annealing and tabu search.

The rest of this study is organized as follows: In Section 2, we present a brief literature review of interterminal transport in the port area, interterminal truck routing optimization, and reinforcement learning (RL) for routing optimization. Section 3 presents the problem under analysis. Section 4 discusses the proposed method, which utilizes RL to tackle the problem of empty truck trips. Section 5 presents the experimental results of our method and compares it with other approaches. Finally, the conclusions are drawn in Section 6 as well as future research ideas.

## 2. Literature Review

### 2.1. Interterminal Transport in Port Area

ITT plays an essential role in most large ports around the world with multiple terminals. Efficient ITT operations significantly contribute to port competitiveness. Therefore, the design of efficient ITT operations in the future will pose a considerable challenge for many large seaports. Hu et al. [2] conducted a comprehensive literature review related to the planning of interterminal transport in port areas and the hinterland. The research was motivated by two factors: the limited research related to the integrated planning of ITT between seaport and inland terminals and the limited studies that summarized the research findings and identified the directions for future research regarding ITT. High-level overall planning is a challenge in major ports around the world because they typically have multiple terminals and facilities, which are often operated by different operators. Therefore, integrated planning is critical for providing effective services. The authors attempt to identify three significant factors for an efficient ITT system: the objectives that should be achieved in ITT system planning, the involvement of the actors, and the methodologies that can be used to support the decision-making process. 

Two planning problems should be tackled to achieve the objective in ITT system planning: The strategical planning problem, and tactical and operational planning problem. In the strategical planning problem, the proper design of the terminal layout and choosing the right ITT fleet can affect the port ITT demand and cost. The increasing number of containers entering and leaving container terminals needs to be handled and accommodated adequately. The new layout of the container terminal that makes container transfer between landslide and seaside faster, cheaper, and more efficient is required. Gharehgozli et al. [8] conducted an extensive literature review on the transition of terminal layout designs from traditional to automated and future container terminals. The author’s study is critical for terminal operators that are looking for technologies and methodologies that can help them to improve their efficiency while at the same time will also increase their terminal capacity and reduce the environmental impacts of their operations. Ottjes et al. [9] performed a comparison of three-terminal configurations: compact configuration, dedicated configuration, and combined configuration. Firstly, two configurations, compact and dedicated configurations, are two extreme conditions where all terminals are connected with multiple modalities or a single modality. The combined configuration represents the planned layout of the Rotterdam Maasvlakte terminals. From their simulation, the results show that the number of ITT vehicles that is used in the dedicated configuration is two times larger than in the compact configurations. Evers and De Feijter [10] investigated the options between centralized and decentralized feeder ship service to reduce the ship service time. The results of their study showed that the centralized service can reduce the vessel average in-port time while using the same number of ITT vehicles. The utilization of more than one transport mode (intermodal transport) to transport the necessary container from one location to another has contributed to additional advantages and limitations that will also contribute to ITT system performance. Generally, road transport is commonly used because of its flexibility, but the study of Gharehgozli [7] found that AGVs and MTSs result in higher operational cost savings due to reduced labor costs. The other transport modes have a trade-off in terms of costing, handling, and waiting time. Railway has a lower transport cost compared to road transport and a higher transport speed compared to waterway transport. However, rail transport requires complicated and long handling time, leading to high ITT costs [11]. In tactical and operational planning problems, proper planning is intended to minimize the ITT timespan or ITT-related cost. Several operations that may affect ITT timespan are transporting, handling, storing, etc. The potential cost relates to the ITT operation that is vehicle fuel consumption cost, vehicle hiring cost, handling fee, storage cost, lateness delivery cost, etc. Kostrzewski and Kostrzewski [12] conducted a thorough analysis of a specific intermodal transport unit, that is, reach stacker. The value obtained from the author’s study is very critical for the analysis, simulation, and numerical models of the intermodal freight terminals, which should consider when minimizing the ITT timespan. Some research focuses on allocating a deep-sea vessel to several different terminals in order to reduce the extra storage costs. This research is crucial because when a deep-sea vessel visits a terminal, some containers should be discharged and loaded onto another vessel in another terminal. At the same time, some export containers terminal in the other terminal must be loaded onto this vessel. Without proper vessel allocation, containers will be stored in the yard and wait for the ITT, and it will lead to the additional storage cost. Hendriks et al.’s [13] study focused on the berth allocation problem to achieve two objectives, which are to balance the quay crane workload over terminals and overtime and to minimize the amount of inter-terminal container transport. Many researchers have also studied the routing of ITT vehicles. Caballini et al. [14] studied the rail cycle in port and proposed a planning approach to minimize the queuing time in multiple yards. Li et al. [15] aimed to reduce the travel time in port by considering the possible disturbance such as terminal equipment failure and sudden closing of terminals. Hu et al. [16] and Hu et al. [17] focused on integrating ITT within the port area by considering the transshipment operations and railway timetable. The model proposed by the authors can help terminal operators to schedule the ITT fleet and RMGs in terminals. The results of their research showed that more flexible ITT connections and a flexible railway timetable can improve the transport performance of containers that are delivered to the hinterland.

Heilig and Voß [18] presented an extensive overview of ITT-related research to reflect the current state of ITT research. There are many factors that influence the productivity and efficiency of ITT as well as its economic and environmental implications. ITT can be considered as a large and complex freight transportation network connecting all terminals and other shared port facilities. Therefore, it requires a higher level of coordination because its internal and external container flows and handling activities must be coordinated by at least two separate parties. Extensive studies need to be conducted to obtain a proper understanding of ITT operations and to reduce its operational costs while strengthening long-term competitiveness. From the author’s study, most ITT-related research works focus on modeling and evaluating different ITT configurations and concepts using optimization and simulation approaches, or both, which are heavily dependent on data input in practical applications. Innovative technologies, alternative ITT systems, and interdisciplinary research should be conducted to solve the future challenges of ITT. An example of these innovative technologies is developing an information system that provides real-time data exchange for decision support, collaboration among stakeholders, information sharing, and improvement of the planning process. The study also suggested future strategies for a more integrated decision support system that facilitates planning, interaction, and collaboration among port stakeholders to improve ITT operations in the economic and environmental aspects.

Duinkerken et al. [19] proposed a rule-based simulation model to evaluate three different transportation systems: multi trailer systems (MTSs), automated guided vehicles (AGVs), and automated lift vehicles (ALVs) in Rotterdam’s Maasvlakte port area. The simulation experiments provide essential insights into three different characteristics of these transportation systems, including an evaluation of the performance and nonperformance of ITT, utilization of transport vehicles with and without advanced planning, and cost analysis to support investment decisions. Tierney et al. [3] presented an integer programming model to minimize container delivery delay by considering significant ITT aspects, including multiple vehicle types, loading/unloading times, traffic congestion, and arbitrary terminal configurations. The proposed model helps in analyzing ITT requirements for new and expanding seaports. The authors used a real example from Maasvlakte and the port of Hamburg to show the benefits of the proposed time-space mathematical model for supporting decisions not only to configure the transport vehicles but also to optimize vehicle routes and container flows in the ITT networks.

### 2.2. Interterminal Truck Routing Optimization

The main tasks of ITT operations are the efficient collection and delivery of containers at the desired terminals. ITT is a critical factor for performance-associated supply chains and impacts the overall reputation of the ports. From an operational perspective, several aspects need to be considered to improve ITT operations. These include the selection of transport modes (e.g., trucks, barges, and trains), coordination of parties involved in ITT operations, and management of external factors influencing the performance of ITT operations (e.g., traffic congestion, equipment breakdown, and truck delays). Additionally, Heilig and Voß [18] found that vehicle routing plays an essential role in coordinating IT flows to reduce operational costs and environmental impact as well as improve customer satisfaction. However, studies on vehicle routing in the ITT context are still limited. Stahlbock and Voβ [20] presented a comprehensive survey on arising routing problems in the container terminal domain. For horizontal transport at the landside, specifically for transporting containers using a truck, route optimization is proposed because it is considered flexible and fast. Furthermore, online optimization is necessary because the situation changed dynamically in the real world. Jin and Kim [21] studied truck routing in Busan port using delivery time windows. They proposed a mathematical model to maximize the profits of a multitrucking company by considering the truck usage cost and delay penalty. Heilig et al. [22] extended ITT truck routing optimization by considering the environmental aspect, thus minimizing truck emissions. The proposed multi-objective model aimed to reduce fixed vehicle hiring costs, vehicle traveling costs, lateness delivery penalties, and emission costs. Heilig et al. [23] presented the interterminal truck routing problem (ITTRP) as a novel vehicle routing problem. It incorporated two greedy heuristics and two-hybrid simulated annealing (SA) algorithms to improve the port actor’s coordination by minimizing truck drayage costs. Their research also introduced the use of a cloud-based centralized communication system and a mobile application to optimize truck routing online. The truck routing optimization considered the fixed vehicle costs, variable vehicle operating costs, and penalty cost for late delivery.

### 2.3. Reinforcement Learning for Route Optimization

To the best of our knowledge, the use of the reinforcement learning-based approach to tackle the vehicle routing problem (VRP) in the context of ITT is still limited. Mukai et al. [24] adopted and improved the native Q-learning, one of the reinforcement learning (RL) algorithms, to optimize the route of on-demand bus systems. In the on-demand bus system, the travel routes for the buses were not determined in advance. The buses pick up customers door-to-door when required. By improving the updated process of the Q values, the results showed the effectiveness of the proposed method in addressing the problem. Similar to Mukai et al. [24], Jeon et al. [25] also implemented the Q-learning algorithm to identify routes with the shortest travel time for AGVs in port terminals. They determined the shortest-time routes inclusive of the expected waiting times instead of the simple shortest distance routes, which are usually used in practice. The waiting time must be estimated accurately to determine the total travel time. The estimation of the waiting time was achieved using the Q-learning technique and constructing the shortest time routing matrix for each given set of positions of the quay cranes. The results showed that the travel time can be reduced by 17.3% using the learning-based routes instead of the shortest distance routes. Kalakanti et al. [26] proposed a reinforcement learning solver for the vehicle routing problem (RL SolVer Pro). They considered the optimal route learning problem as a Markov decision process (MDP). The two-phase solver was used with geometric clustering to overcome the RL curse of dimensionality. Their simulation results showed that the proposed method obtained better, or similar results compared to the two best-known heuristics, namely, Clarke–Wright savings and sweep heuristics. Ye et al. [27] proposed a novel deep reinforcement learning-based neural combinatorial optimization strategy to develop vehicle routing plans for city-size transportation networks. The authors transformed the online routing problem to a vehicle tour generation problem and developed a structural graph embedded pointer network to produce the tours iteratively. To assess the proposed strategy, the authors conducted comprehensive case studies on a real-world transportation network and dynamic traffic conditions in Cologne, Germany. The simulation results showed that the proposed approach can significantly outperform conventional strategies with limited computation time in both static and dynamic logistic systems.

In this study, we employ Deep Q-Network (DQN), because our case study has a large state-action space (approximately 576,000 combinations of all states and actions). The use of a tabular RL fashion such as Q-Learning to store the value function or policy for a large state-action space is considered inefficient [28]. The utilization of the deep neural network in deep Q-networks as a function approximation aims to make generalizations from examples of a function to construct an approximate of the entire function [29]. The details of the proposed DQN design are discussed in Section 4.

## 3. Problem Description

The ITTRP used in this study is similar to the one presented by Heilig et al. [23]. A homogeneous vehicle pickup and delivery problem is created using time windows by considering the maximum availability of the truck working hours, penalties for delayed orders, time windows of locations, and due dates of transport orders. The proposed ITTRP version does not consider external trucks, and the site used in our experiment covers only five container terminals. As defined, ITTRP is the process of moving containers between facilities (e.g., container terminals, empty container depots, value-added logistics) within a port. Therefore, we have sets of locations, trucks, and customers represented by *L*, *T*, *C*, respectively, where each customer has a set of requesting orders $R^{k}\in \left\{j_{1}^{k},\;j_{2}^{k},\ldots ,\;j_{\left|R^{k}\right|}^{k}\right\}$, where *k* is the index of the customer. The set of all requesting orders is $O\;=\;\;\cup_{k\in C}\;R^{k}$. Furthermore, each order, $j_{i}^{k}$, k∈C, $i\in R^{k}$, has a given origin $o\left(j_{i}^{k}\right)\in L$ and destination $d\left(j_{i}^{k}\right)\in L$. The subsets of the origin and destination are denoted as $L^{s}$ and $L^{d}$, respectively. Each order must be served by considering the service times at the pickup (i.e., origin) and delivery (i.e., destination) locations, which are denoted as, $S_{\left(j_{i}^{k},\;o\left(j_{i}^{k}\right)\;\right)}$ and $S_{\left(j_{i}^{k},\;d\left(j_{i}^{k}\right)\;\right)}$, respectively. An associated order penalty $pn_{j_{i}^{k}}$ is applied if the transport order cannot be completed before a given due date $dd_{j_{i}^{k}}$. Additionally, each truck t∈T has an initial position, $ip_{t}$, a maximum number of working hours, $mx_{t}$, a prefixed cost for using a truck, $cs_{t}$, and variable costs per hour, $hr_{t}$. The objective of this problem is to minimize the costs related to the use of trucks, which can be illustrated by the following objective function:

(1) $$minimize\;\left(\sum_{t\in T}x_{t}.cs_{t}+\sum_{t\in T}tm_{t}.hr_{t}+\sum_{o\in O}y_{o}.pn_{o}\right)$$

where $x_{t}$ is equal to 1 if truck t is hired, 0 otherwise; $y_{0}$ is equal to 1 if the order *o* is performed after its due date and 0 otherwise. The service time required by a truck *t* for performing all its assigned transport orders is denoted as $tm_{t}$. A solution for the ITTRP must satisfy the following feasibility restrictions:

(1) Each transport order must be performed.
(2) Each transport order must be performed by a single truck.
(3) Each transport order $j_{i}^{k}$ is ideally performed before its due date, taking into account the service time at the destination locations. The penalty cost $pn_{o}$, o∈O, will be charged if the truck cannot complete a transport order within its due date.
(4) Each route begins at the starting position of the truck. In our case, the initial position of all trucks is at a terminal one, and the next starting point of each truck is the destination location of the latest order served by the truck. The time and cost required for moving trucks from the initial location to the order origin (for first-time order assignments) are not considered in the objective function.
(5) Pickup and delivery operations are considered as pairing constraints in the transport order.
(6) The pickup vertices are visited before the corresponding delivery vertices (precedence constraints).
(7) Each location $l\in L^{s}\in L^{d}$ has a given availability time window; hence, the trucks can only arrive at the origin and destination based on their given time windows. When the trucks arrive in advance, they must wait until the location operation time starts and the location is ready for pickup or delivery activities.

Moreover, we consider the following assumptions:

(1) The truck speed and distance between two terminals to calculate the travel time as well as service time and time windows are known in advance.
(2) The fee for performing transport orders, fixed costs, and variable costs is known in advance.

## 4. Proposed Method

### 4.1. Reinforcement Learning

**Figure 2:**
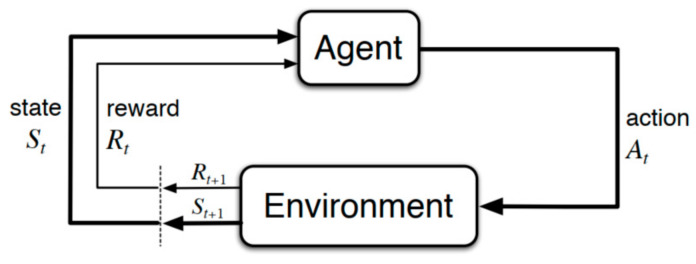
Agent–environment interaction in RL.

RL is a machine learning technique that allows an agent to learn by performing trial-and-error-based interactions with the environment. RL is different from the other machine learning techniques: supervised learning and unsupervised learning. In supervised learning, there is a mapping between the input and output, while in unsupervised learning, unlabeled input data are utilized to discover unknown relationships or structure within them. Unlike supervised and unsupervised learning, RL is defined by characterizing a learning problem, not by characterizing the learning method. RL uses rewards which are obtained when interacting with the environment, as a signal for positive and negative behavior. The goal of supervised learning is to find similarities and differences between data points, whereas the goal of RL is to find the best action for maximizing the total cumulative reward of the agent. Figure 2 shows the agent–environment interaction. An RL agent takes action in discrete time steps from a set of available actions and receives a reward for choosing the best action or penalty for the bad action. A reward is a numerical measure of the goodness of an action that depends on the state transition. After performing an action, the agent proceeds to the next state. The agent interacts continuously with the environment to learn the best action for a particular state by collecting rewards and penalties over time.

The RL problem is defined in the form of a Markov decision process (MDP), a classical formulation of sequential decision making, in which both immediate and future rewards are considered. An MDP is described by a tuple $M\;=\;(S,\;A,P(s_{t+1},\;r|s,a),\;R,\gamma )$, where:

- s∈ S is a set of all possible states describing the current situation or condition of the environment.
- a∈ A is a finite action space—a set of all available actions of the agent.
- $P(s_{t+1},\;r|s,a)$ denotes the probability of transitioning to $s_{t+1}$ and receiving a reward, r, given s∈ Sa∈ A as follows: $P\left(s_{t+1},\;r|s,a\right)\;=\;\mathrm{Pr}\{S_{t+1}\;=\;\;s_{t+1},\;R_{t}\;=\;r\;|\;S\;=\;s,\;\;A\;=\;a\}$
- R∈ℝ is the expected reward received from the environment after the agent performs action *a,* at state *s*.
- $\gamma \in \left[0,\;1\right]$ is a discount factor representing the discounted future returns.

RL consists of two essential components: the agent and the environment. In our case study, the agent is a truck which interacts with the environment by choosing an order from a given set of transport orders at a particular time. As illustrated in Figure 2, the state of the environment at time step *t* is denoted as $s_{t}$. Subsequently, the agent examines $s_{t}$ and executes a corresponding action $a_{t}$. The environment then changes its state $s_{t}$ to $s_{t+1}$ and gives the agent a numerical reward $r_{t+1}$. The goal of the agent is to find the optimal policy π, S x A, which maximizes the expected return from each state $s_{t+1}$. The expected return $R_{t}$ at time *t* is defined as follows:

(2) $$R_{t}=E[\overset{\propto }{\underset{k=0}{\sum }}\gamma^{k}r_{t+k}]$$

where γ is the discount factor that determines the tradeoff between immediate and future rewards. The expected return in the Q-learning algorithm after an agent executes an action is defined as follows:

(3) $$Q^{\pi }\left(s,a\right)\;=\;E[R_{t}\;|s_{t}\;=\;s,a]$$

where $Q^{\pi }\left(s,a\right)$ is the discounted future reward when an agent performs action *a*, in state *s*. The maximum action value for state *s* and action *a,* achievable by any policy, is defined as follows:

(4) $$Q^{*}\left(s,a\right)\;=\;max_{\pi }Q^{\pi }\left(s,a\right)$$

The detailed design of each state, action, and reward is discussed in the next subsection.

#### 4.1.1. State Representation

In the ITTRP problem, an agent (a truck) observes the current state, *s*, from the environment at time *t*. A truck must consider the available transport orders and the truck’s current position at a particular time when picking an order. Moreover, the available transport orders can have one or more transport order characteristics. These define two factors: the distance between the current truck position and transport order origin and the time gap between the current time and the transport order due date. 

**Figure 3:**
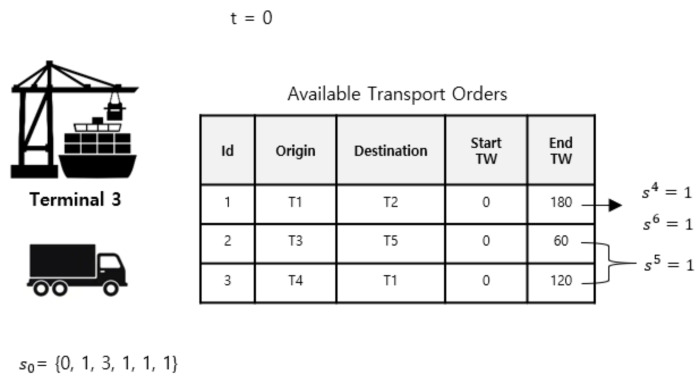
Example of state at time step t = 0.

There are three categories of transport order characteristics: transport orders that have an order origin similar to the truck current position (OC1), transport orders that have the nearest due date (OC2), and transport orders that have the farthest due date (OC3). The characteristics of the last two transport orders are determined by calculating the gap between the current time and the end time window of the available transport orders. If the difference is more than two hours, then the transport order has the farthest due date; otherwise, the transport order has the nearest due date characteristics. Figure 3 illustrates an example of a state at time step t = 0, which shows that the truck is currently located at container terminal 3, and there are three available transport orders with three order characteristics. Based on the aforementioned condition, the state at time step t = 0 is declared as *s*_0_ = {0, 1, 3, 1, 1, 1}.

A set of states, *S*, in our case study, consists of six elements. At time step *t*, $S\;=\;{\left\{s^{1},s^{2},s^{3},s^{4},s^{5},s^{6}\right\}}_{t}$, where:

- $s^{1}$ represents the current time in minutes. The value has a range of 0–1440 because it represents a 24 h range in minutes.
- $s^{2}$ represents the available transport orders. The value is one if there is at least one transport order available, otherwise, it is zero.
- $s^{3}$ represents the position of the truck. In our case, we consider five terminal locations. The value of this element has a range of one to five.
- $s^{4}$ indicates the presence of transport orders that have an order origin similar to the truck’s current position. The value is zero or one.
- $s^{5}$ indicates the presence of transport orders that have the nearest due date. The value is zero or one.
- $s^{6}$ indicates the presence of transport orders that have the farthest due date. The value is zero or one.

#### 4.1.2. Actions

An agent can choose a possible action at each time step *t*. In our case, a set of actions, A, consists of five elements, $A\;={\left\{a^{1},a^{2},a^{3},a^{4},a^{5}\right\}}_{t}$ where:

- $a^{1}$: represents idle
- $a^{2}$: choose random order
- $a^{3}$: choose order with OC1 characteristics
- $a^{4}$: choose order with OC2 characteristics
- $a^{5}$: choose order with OC3 characteristics

The goal of the agent is to learn a policy π that maximizes the cumulative reward. By definition, policy π is a function that defines the probability of an action *a* to get chosen in state *s*: S → p(A = a|S). In our case, policy π is a function from states to actions and is represented as a deep neural network. Given the current state values, the agent can determine the best action value through the policy.

#### 4.1.3. Reward Function

The reward function design is vital to induce an agent to achieve the goal, which is to maximize the cumulative reward. A reward provides feedback to the agent after performing a chosen action. The ultimate goal of the agent is to find an optimal truck route that has a minimum total cost of using the truck, which includes total travel cost, total empty truck trip cost, and penalty cost. In this study, we propose four reward cases at each time step:

(1) R(*t*) = 0.01, if choose action $a^{1}$ where there is no available transport order.
(2) R(*t*) = 0, if performing an improper action such as:
  a. $TC_{O_{i}}$ ≥ ATC
  b. Choose action $a^{1}$ when there is at least one transport order.
  c. Choose action $a^{2}$ when there is no available transport order.
  d. Choose action $a^{3}$ when order with OC1 characteristics is not available.
  e. Choose action $a^{4}$ when order with OC2 characteristics is not available.
  f. Choose action $a^{5}$ when order with OC3 characteristics is not available.
(3) R(*t*) = 1, if
  a. $TC_{o}$ ≤ ATC, where $TC_{o}$ is the total cost of performing the current transport order, and ATC is the average of the total cost of performing all previous transport orders calculated using the following equation $ATC\;=\;\;\frac{1}{n}\overset{i}{\underset{n\;=\;0}{\sum }}TC_{o_{n}}$.
(4) R(*t*) = 25, if the terminal condition is reached and $STC_{eps_{i}}\leq ASTC$, where $STC_{eps_{i}}$ is the sum of the total cost of performing all transport orders at episode *i,* and ASTC is the average of STC of all episodes.

The first reward case is taken from choosing an idle action when there is no available transport order at the current time. In this case, the reward value is set at a small amount such as 0.01 to prevent the agent from considering it as the best action when it has to be performed many times and makes its reward accumulation exceed the reward accumulation of taking the expected action. The reward function design should also cover how to make an agent avoid undesirable behavior. The reward of taking improper action categorized in the second case was zero. For instance, the agent will obtain a zero reward when taking an idle action while there are available transport orders. The zero rewards for this case give a signal to the agent; taking an idle action is not expected when there are available transport orders. The third and fourth reward cases describe the immediate and delayed rewards, respectively. An agent obtains an immediate reward for every decision in selecting and serving a transport order that produces a total cost less than the average of the total cost of performing all previous transport orders. We formulated the truck routing optimization as an episodic task. The RL training process executes many episodes. The total cost of each episode must be calculated and evaluated to determine if there is a learning improvement of the agent. The simulation process terminates in a specific state. In our case, it ends when all transport orders have been served. The total cost sequence of the served transport orders is calculated at each episode and compared with the total cost of the previous episode. If the current episode’s total cost is less than the previous one, the agent receives a delayed reward.

### 4.2. Deep Q-Network

Deep reinforcement learning (Deep RL) is a combination of deep learning (DL) and RL to deal with high dimensional state and action spaces [29]. In 2015, Mnih et al. [30] proposed a novel structure named the DQN that combines RL and a convolutional neural network (CNN), which proved successful in making an autonomous agent play competently in a series of 49 Atari games. The application of CNN in a DQN is intended to directly interpret the graphical representation of the input state, *s,* from the environment. In this study, we employ a DQN because the state-action space, in our case, is considerably large.

**Figure 4:**
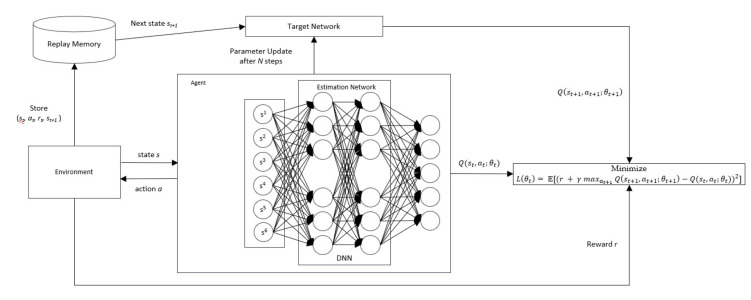
Deep Q-Network structure.

The tabular fashion of Q-learning is challenging to converge in a high-dimensional environment because it must visit all possible state-action pairs infinitely [31]. The use of DNN in DQN acts as a policy approximation function. As illustrated in Figure 4, in the model-free RL, $Q\left(s_{t},a_{t};\theta_{t}\right)$ is represented as an approximator with parameter $\theta_{t}$ that needs to approach the optimal action value, that is:

(5) $$Q^{*}\left(s_{t},a_{t}\right)\approx Q\left(s_{t},a_{t};\theta_{t}\right)$$

where parameter $\theta_{t}$ is learned iteratively by minimizing the following loss function:

(6) $$L\left(\theta_{t}\right)\;=\;E{\left(r+\;\gamma \;max_{a_{t+1}}Q\left(s_{t+1},a_{t+1};\theta_{t}\right)\;-Q\left(s_{t},a_{t};\theta_{t}\right)\right)}^{2}$$

where $r+\;\gamma \;max_{a_{t+1}}Q\left(s_{t+1},a_{t+1};\theta_{t}\right)$ is the target value, $s_{t+1}$ and $a_{t+1}$ represent the state and action at time step *t+1*, respectively. The target value must be replaced with weight $\theta_{t+1}$, which *is updated at every N* step from the estimation network to address the instability issue in the DQN. This leads to the following loss function equation:

(7) $$L\left(\theta_{t+1}\right)\;=\;E{\left(r+\;\gamma \;max_{a_{t+1}}Q\left(s_{t+1},a_{t+1};\theta_{t+1}\right)\;-Q\left(s_{t},a_{t};\theta_{t}\right)\right)}^{2}\;\theta_{t+1}\leftarrow \;\theta \;for\;every\;N\;steps$$

Moreover, the generated samples $\left(s_{t},\;a_{t},\;r_{t},\;s_{t+1}\right)$ are stored in an experience replay memory. These samples are then retrieved randomly from the experience replay and fed into the training process.

## 5. Experimental Results

In this section, simulation experiments are conducted to evaluate the performance of the proposed method. The algorithm was implemented in Python and run on a PC equipped with an Intel^®^ Xeon^®^ CPU E3-1230 v5 of 3.40 GHz and 16 GB memory. We train the DQN for 750 episodes and using 250 files in which each file contains 285 transport order data, as illustrated in Table 1. The number of files used in the training process represents the different variation characteristics of the transport order data, which might occur in the real container terminal. This kind of data variation will make the agent of our DQN learn to find the optimal policy from a different situation. The whole DQN training process took roughly eleven days. The training process of the first file consumed the longest training time, which approximately ran for 119 h, while the training process of the remaining files only needed 40 min on average. This phenomenon occurs because, in the first 750 episodes of the first file, the DQN still forming the learning model from scratch while the next remaining training process took advantage of the trained model from the previous training process.

First, we present the data that were used in this study, such as an example of the transport order data, container movement data, container processing time, as well as the costs and fee of transporting a container. Subsequently, we present the configuration of the proposed method and the other two metaheuristic algorithms. Finally, we present the performance of our proposed method compared to the two different metaheuristic algorithms by showing the results of the three performance parameters, namely, total cost, empty truck trip cost, and computational time.

### 5.1. Data

In this study, we use the transport order data as shown in Table 1, which contains four crucial factors: the order origin, destination, start time window, and end time window of the order. These data are artificially generated by considering the following values range:

(1) order origin (*o*): {T1, T2, T3, T4, T5}
(2) order destination (*d*): {T1, T2, T3, T4, T5}, where *d ≠ o*
(3) start time window (in minutes): {0, …, 1320}
(4) end time window (in minutes): {120, …, 1440}

**Figure 5:**
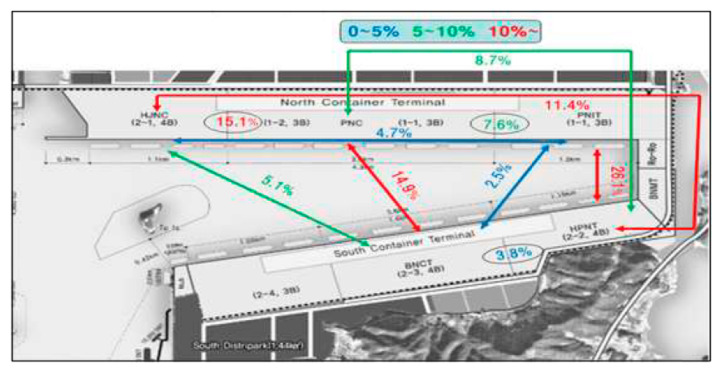
Busan New Port Container Terminal Layout [32].

The generated data are made to mimic the real seaport, in our case, the Busan New Port (BNP), as shown in Figure 5, which has five container terminals and operates for 24 h (1440 min). 

We use the information on the container movement rate between the terminals of BNP, as shown in Table 2, which was published by Park and Lee [32]. By considering this information, the generated data obtains characteristics from the real container terminal in terms of movement rates between terminals.

The other crucial transport-related information is the container processing time, which includes the terminal to terminal travel time, the time required for traffic lights, the gate passing time, and the waiting time for loading/unloading, as shown in Table 3. We also obtain this container processing time from the research paper by Park and Lee [32]. The container processing time is critical because the state in our proposed RL involves the time variable.

Standard costs and fees are required to calculate the truck-related costs in transporting a container. We use the standard published by Jin and Kim [21], which included the truck transport cost, idle cost, delay cost, and revenue per container, as shown in Table 4.

One-time period in Table 4 corresponds to a 15 min time unit. For example, a truck transportation cost of $4/time-period means that the transportation cost is $4 for every 15 min. Both container processing time, and cost and fee are critical for the RL learning environment for the agent to experience the near-real condition of the container terminal.

The variation of the transport order data is essential to evaluate the performance of the proposed algorithm. In this experiment, we use three categories of datasets in terms of the number of transport orders, which are: datasets which are less than 100 orders, between 100 and 200 orders, and more than 200 orders, as shown in Table 5.

### 5.2. Algorithm Configuration

#### 5.2.1. DQN Configuration

DL in this experiment was developed using KERAS library version 2.3. The DL model has six inputs, two hidden layers with nine neurons for each layer, and five outputs. The input is set to six because, in our case, the input of our DQN should accommodate all elements of the state, which is composed of six elements, while the output of our DQN is the number of possible actions. The number of hidden neurons was determined based on [33], which stated that the number of hidden neurons should comply with the following rule-of-thumb:

(1) The number of hidden neurons should be between the size of the input layer and the size of the output layer.
(2) The number of hidden neurons should be 2/3 the size of the input layer, plus the size of the output layer.
(3) The number of hidden neurons should be less than twice the size of the input layer.

All hidden layers use a rectified linear activation function, whereas the output layer uses a linear activation function. 

The DQN was trained using the configuration present in Table 6, using 500 variations of transport data, and each variation running for 1000 episodes. 

#### 5.2.2. Simulated Annealing Configuration

The SA algorithm is a probabilistic method proposed by Kirkpatrick et al. [34], which emulates the physical process in which a metal is heated at a very high temperature and slowly cooled until it reaches a frozen state. The initial temperature (*T_initial_*) of the SA was set to 100 degrees, the cooling rate (α) was calculated using the following equation ${\left(\frac{T_{final}}{T_{initial}}\right)}^{\left(\frac{1}{num.\;of\;iterations\;–\;1}\right)}$, and the stopping criterion was when the temperature (*T_final_*) reached 0.0001 degrees. The highest value of *T_initial_* and the lowest value *T_final_* was chosen to give the SA algorithm adequate time for leaving local optima as states in the [35] study. The Boltzmann probability $P\left(S_{new}\right)\;=\;e^{\left(-\Delta S/T\right)}$ is used to accept the new solution. If this probability is higher than a random value between 0 and 1, then the new solution *S_new_* is accepted. The number of iterations for the SA algorithm was set based on the study of [36], which states that if the candidate size is more than 200, then the number of iterations should be set to 100,000. In our case study, the candidate size is 285, and then 100,000 iterations were determined for the number of iterations.

#### 5.2.3. Tabu Search Configuration

Tabu search (TS) is a metaheuristic method that guides a local heuristic search procedure to explore the solution space to escape the trap of the local optimal. The basic form of TS was proposed by Glover [37]. The TS algorithm is considered a highly efficient search algorithm. The memory mechanism and tabu criteria in the TS algorithm can avoid circuitous search conditions. The aspiration criteria used in TS can also ensure a diversification search and obtain the global optimum. In our experiment, the length of the tabu list is seven. The number of iterations for the TS algorithm is 100,000 iterations.

The neighborhood search method is used for both SA and TS; we use two transformation rules for generating neighbors as proposed by Tan [38]. The first transformation rule is to exchange routes within a vehicle, and the second is to exchange routes between two vehicles. The new solution from the process of generating neighbors is evaluated to meet the main objective function, which is the minimization of the total cost that includes total travel cost, total empty truck trip cost, and penalty cost. 

### 5.3. Results

**Figure 6:**
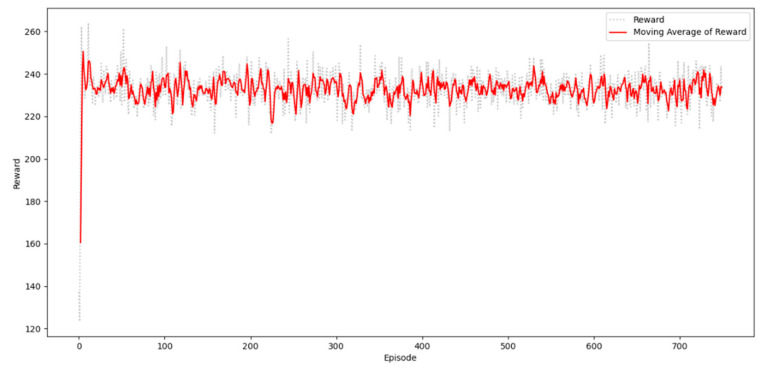
DQN performance on finding the optimal truck route.

Figure 6 shows the DQN performance on finding the optimal truck route. The *x*-axis represents the episodes, and the *y*-axis represents the cumulative rewards per episode. From the 750 episodes, the rewards show a significant increasing trend. The DQN can quickly adapt to the environment within the first 100 episodes.

**Figure 7:**
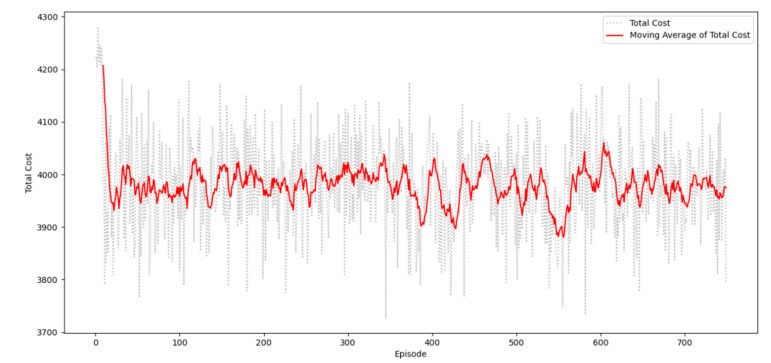
The truck route’s total cost per episode.

In this study, the optimal truck route is defined by evaluating the required total cost. Figure 7 shows the decreasing trend in the total cost per episode. The *x*-axis represents the episodes, and the *y*-axis represents the truck route’s total cost per episode. The movement of the total cost is consistent with the reward trend. It decreases when the reward increases.

Each experiment was tested 30 times, and the average values of the three performance parameters (total cost, empty truck trip cost, and computational time) were obtained. Table 7 lists the abbreviations of the parameters used in this study.

As shown in Table 8, the DQN exhibits better performance than SA because it gives the best results on all three datasets: DC1-35, DC1-89, and DC1-173, in terms of TC and ETTC. The SA algorithm gets the best results for the DC2-116 and DC3-285 datasets under the same performance parameters. In terms of CT, SA tends to have a linear CT when the number of data points increases. Unlike the DQN, the CT for DC2-116 shows nonlinearity. Based on our evaluation, this condition occurs when the test dataset used in the testing has similar characteristics to the dataset used for training; therefore, the DQN only requires a short time to produce results.

Table 9 shows that the TS exhibits considerable performance in terms of TC and ETTC for the DC1-35, DC2-116, and DC3-285 datasets. The DQN obtains the best results for the DC1-89 and DC2-173 datasets in the same performance parameters. In terms of CT, the TS has a similar CT characteristic with SA on average, whereas DQN tends to have a faster CT than TS. The performance gap presented in Table 10 shows the level of improvement among the algorithms. Based on the performance gap table, we can conclude that the DQN yields up to 9%, 22.9%, and 80% better results for TC, ETTC, and CT, respectively. Although the DQN does not entirely show better results across all datasets, we observe that it achieves a comparable solution quality.

## 6. Conclusions

Many major ports around the globe are affected by the increasing volume of global containerized transport. This condition necessitates port authorities to improve port performance by optimizing productivity and efficiency. ITT plays an essential role in large ports with multiple terminals. Without proper planning, ITT can be one of the sources of significant inefficiency that will negatively influence the overall port performance. The study of route optimization related to ITT is still limited. Moreover, the use of the reinforcement learning approach for route optimization is scarce. In this study, we proposed a DQN to provide a near-optimal interterminal truck route and to meet the main objective of ITT, which is to minimize the total costs related to the use of trucks. We designed a custom-specific state and action so that the agent of the RL can learn the best decision in producing a truck route with minimum total costs. We conducted computational experiments to verify the performance of the proposed method. We chose the simulated annealing and tabu search algorithms as the baseline for comparison. In general, the proposed DQN can provide a feasible solution within a short computational time. Compared to the two-baseline algorithms, the DQN exhibited a considerable quality of performance and solution. The proposed method can be applied to solve the real-world case by considering the following requirements:

(1) the real-world case should have similar characteristics as our case study, which consists of five container terminal, 24 h of the operational working hour, and only for homogeneous vehicles.
(2) the transport order data must have four essential elements: order origin, destination, start time window, and end time window.

Those requirements can also be the limitations of our proposed method. The method we proposed requires some modifications if it is to be applied to other cases that have different characteristics. Therefore, techniques that can solve the general problems of ITT are still open for future research. Some challenging issues still need to be addressed in future research. One of them is the development of a stable DQN that requires less training data and training time but can provide a feasible solution for the general case of ITT.

## Figures and Tables

**Table 1:** Example of transport order data.

Origin	Destination	Start-Time Window	End-Time Window
T1	T2	60	480
T4	T5	30	360
T3	T1	120	860

**Table 2:** Container movement rate between terminals [32].

From/To	PNIT	PNC	HJNC	HPNT	BNCT
**PNIT**	-	7.6%	4.7%	26.1%	2.5%
**PNC**	7.6%	-	15.1%	8.7%	14.9%
**HJNC**	4.7%	15.1%	-	11.4%	5.1%
**HPNT**	26.1%	8.7%	11.4%	-	3.8%
**BNCT**	2.5%	14.9%	5.1%	3.8%	-

**Table 3:** Estimated container processing time per move [32].

	Terminal to Terminal Time (min)	The Time Required for Traffic Lights (min)	Gate Passing Time (min)	Waiting Time for Loading/Unloading (min)	The Time Required Per Move (min)
PNIT–PNC	2.85	0	0	30	33
PNIT–HJNC	11.35	8	1	30	50
PNIT–HPNT	4.92	4	1	30	40
PNIT–BNCT	11.3	8	1	30	41
PNC–HJNC	5.1	2	1	30	38
PNC–HPNT	10.75	10	1	30	52
PNC–BNCT	5.50	11	1	30	48
HJNC–HPNT	11.62	12	1	30	55
HJNC–BNCT	13.8	13	1	30	58
HPNT–BNCT	4.5	1	1	30	37

**Table 4:** Costs and fee [21].

	Unit	Value
Truck transportation cost	$/time period	4
The operation cost of an idle truck	$/12 time period	0.001
Delay cost	$/container/time period	5
Revenue per container	$/container	25

**Table 5:** Datasets for experiment.

Dataset ID (DID)	Dataset Category	Number of Order
DC1-35	1	35
DC1-89		89
DC2-116	2	116
DC2-173		173
DC3-285	3	285

**Table 6:** Hyperparameters for the training.

Hyperparameter	Value
Num. of episodes	750
Batch-size	32
Replay memory	100,000
Discount factor γ	0.99
Learning rate α	0.001
Epsilon decay ϵ	0.05

**Table 7:** Parameter abbreviations.

Parameters	Abbreviation
Average Computational Time (in seconds)	Avg CT
Best Computational Time (in seconds)	Best CT
Average Total Cost (in $)	Avg TC
Minimum Total Cost (in $)	Min TC
Average Empty Truck Trip Cost (in $)	Avg ETTC
Minimum Empty Truck Trip Cost (in $)	Min ETTC

**Table 8:** Performance comparison between DQN and SA.

	DQN						SA					
DID	Min TC	Avg TC	Min ETTC	Avg ETTC	Min CT	Avg CT	Min TC	Avg TC	Min ETTC	Avg ETTC	Best CT	Avg CT
DC1-35	**291.6**	**310.77**	**84.13**	**103.3**	32.67	56.59	316.66	341.58	109.2	128.01	**23.68**	**51.42**
DC1-89	**790.66**	**828.03**	**263.2**	**299.53**	108.41	148.4	860.26	905.4	332.79	346.44	**101.92**	**134.86**
DC2-116	1054.66	1096.02	367.99	409.35	**25.05**	**54.57**	**1011.06**	**1052.59**	**324.39**	**365.92**	118.93	157.14
DC2-173	**1619.73**	**1660.12**	**594.26**	**633.18**	**91.53**	**146.05**	1722.93	1819.7	684.13	706.85	167.43	223.91
DC3-285	2720	2787.11	1032	1099.11	**189.73**	**315.13**	**2707**	**2746.62**	**1019.06**	**1058.62**	245.87	386.54

**Table 9:** Performance comparison between DQN and TS.

	DQN						TS					
DID	Min TC	Avg TC	Min ETTC	Avg ETTC	Min CT	Avg CT	Min TC	Avg TC	Min ETTC	Avg ETTC	Best CT	Avg CT
DC1-35	291.6	310.77	84.13	103.3	32.67	56.59	305.2	306.29	97.73	**98.82**	**24.14**	**53.44**
DC1-89	**790.66**	**828.03**	**263.2**	**299.53**	108.41	148.4	845.86	849.59	318.39	322.12	**104.6**	**138.28**
DC2-116	1054.66	1096.02	367.99	409.35	**25.05**	**54.57**	**975.73**	**984.23**	**289.06**	**297.56**	125.34	158.36
DC2-173	**1619.73**	**1660.12**	**594.26**	**633.18**	**91.53**	**146.05**	1687.59	1693.17	662.13	667.65	185.53	231.18
DC3-285	2720	2787.11	1032	1099.11	**189.73**	**315.13**	**2649.46**	**2667.45**	**961.46**	**979.45**	249.68	390.58

**Table 10:** Performance gap between DQN, SA, and TS.

	GAP (%)											
	DQN vs. SA						DQN vs. TS					
DID	Min TC	Avg TC	Min ETTC	Avg ETTC	Min CT	Avg CT	Min TC	Avg TC	Min ETTC	Avg ETTC	Best CT	Avg CT
DC1-35	**7.91**	**9.01**	**22.95**	**19.3**	−27.51	−9.13	**4.45**	−1.44	13.91	−4.33	−26.1	−5.56
DC1-89	**8.09**	**8.54**	**20.91**	**13.54**	−5.98	−9.12	**6.52**	**2.53**	**17.33**	**7.01**	−3.51	−6.81
DC2-116	−4.13	−3.96	−11.84	−10.6	**78.93**	**65.27**	−7.48	−10.19	−21.44	−27.3	**80.01**	**65.54**
DC2-173	**5.98**	**8.76**	**13.13**	**10.42**	**45.33**	**34.77**	**4.02**	**1.95**	**10.25**	**5.16**	**50.66**	**36.82**
DC3-285	−0.47	−1.45	−1.25	−3.68	**22.83**	**18.47**	−2.59	−4.29	−6.83	−10.88	**24.01**	**19.31**
